# Safeguarding Victims of Domestic Abuse During COVID-19: Survivor Perspectives

**DOI:** 10.1177/10778012251348435

**Published:** 2025-06-18

**Authors:** Gayatri Nambiar-Greenwood, Khatidja Chantler, Lis Bates, Michelle McManus, Margaret Struthers, Debbie Thackray, Emma Ball

**Affiliations:** 1 School of Nursing and Public Health, 5289 Manchester Metropolitan University, Manchester, UK; 2 Centre for Policing Research and Learning, Open University, Milton Keynes, UK; 3 School of Justice Studies, Liverpool John Moores University, Liverpool, UK

**Keywords:** COVID-19, safeguarding, domestic abuse, multiplier effect, trauma, minoritization

## Abstract

This study presents qualitative interviews with 15 victim-survivors of domestic violence and abuse (DVA) and quantitative analysis of police data in England. It explores survivors’ experiences accessing services during lockdown, identifying themes such as COVID-19-specific challenges, mental health, no recourse to public funds, child impact, cultural bereavement, service responses, and survivor-led recommendations. Key policy implications include improving police ethnicity recording, examining how ethnicity influences risk assessments and outcomes for minoritized victims, and providing culturally appropriate, trauma-informed services. The study calls for state-level preparedness for DVA responses during crises and addressing the structural harm caused by no recourse to public funds policies.

## Introduction

Crises in public health have repeatedly exposed increased experiences of Domestic Violence and Abuse (DVA). Previous global epidemics, such as Ebola, Zika, and Cholera uncovered an associated intensification of intimate partner violence (IPV) and DVA (UN Women et al., 2020). Measures to reduce transmission of infection during COVID-19 lockdowns were necessary but potentially increased the risk of DVA (Usta et al., 2021). Simultaneously, accessing support became more difficult with slowing down or closure of potential support pathways. Increased financial pressures, increased proximity of family members in the same household, isolation, increased burden of domestic labor, and caring responsibilities for women remained potential risk factors for increased rates of DVA. Victim-survivors of DVA had less opportunity to disclose abuse to professionals or to seek support from family and friends (Women's Aid, 2020a). During the first phase of lockdown, in March 2020, in the United Kingdom alone, domestic homicides rose 50% since the lockdown, with a surge in calls to a national domestic abuse helpline (The Guardian, 2020). A survey at this time showed 67% of victim-survivors said that domestic abuse worsened in lockdown (Women's Aid, 2020a, 2020b).

Globally, early emerging pandemic evidence from China, Italy, France, and the United Kingdom indicated DVA rates increasing by 30% during COVID-19 (WHO, 2020). Pre-COVID-19, accessing DVA support in the United Kingdom was already compromised by austerity policies affecting the domestic abuse Non-Governmental Organisation (NGO) sector, and other related services such as mental health services and adults and children's social care (Sanders-McDonagh et al., 2016). There was also evidence that, to ration services, statutory agencies had increased the threshold at which services were provided (Devaney, 2019). These challenges were exacerbated with lockdown (SafeLives, 2020), during different restrictive measures, and even as lockdown eased, Black women and those who shared intersecting identities and immigration status were subject to prejudice, discrimination, and oppression (minoritized), and those with children, continued to struggle (Kaukinen 2020).

Further, those who have no recourse to public funds (NRPF)—an immigration rule that severely restricts access to support services and welfare continues to be a long-standing barrier for Black and minoritized women accessing DVA support (Anitha, 2011; Burman and Chantler, 2005; Banga & Roy, 2020; Women's Aid, 2020b).

DVA in England and Wales is defined as “any incident or pattern of incidents of controlling, coercive or threatening behavior, violence or abuse between those aged 16 or over who are or have been intimate partners or family members regardless of gender or sexuality” (Home Office, 2013:1). This definition stands in the recent Domestic Abuse Act 2021 (Home Office, 2021). Its broad definition encompasses physical, sexual, emotional, financial, and economic abuse, stalking, and coercive and controlling behavior. Where DVA is perpetrated by family members, we use the term adult family abuse to distinguish it from IPV.

This research was established to inform policy developments in England, for safeguarding against DVA, particularly for those silenced, isolated, and not deemed as “high” risk within COVID-19 restricted safeguarding practice. This enabled the identification of challenges as well as best practices within the COVID-19 pandemic with implications for other future national emergencies. As such, our study focused on two knowledge gaps centered on the dual pandemics of COVID-19 and Violence Against Women and Girls (VAWG):

1. A lack of knowledge around the impact of the pandemic on Black and minoritized women's experiences of DVA. Globally, there was recognition that pandemics heightened both, the risk of DVA victimization and structural inequalities (WHO, 2020). As Black and minoritized women experience multiple structural/racialized inequalities, it increases their vulnerability at multiple intersecting levels (Banga & Roy, 2020). Taking into consideration the awareness that marginalized groups in society were particularly vulnerable during the COVID-19 pandemic, we specifically wanted to investigate the experiences of Black and minoritized women in receiving support during the pandemic. The purpose is concretized in some of the following research questions. It was crucial to understand interagency processes for the identification of DVA, referral mechanisms, assessment of thresholds for intervention and methods of working with victim-survivors in the pandemic context.
2. A lack of victim-survivor voices in COVID-19 DVA research. These perspectives are key to informing future best policy and practice. At the time we carried out this research, this type of data was missing in COVID-19 DVA research.

As such, the research questions included:

- What were the experiences of victim-survivors accessing support during COVID-19? and,
- In particular, how did this impact on Black and minoritized victim-survivors and their children?
- What were police responses to Black and minoritized women pre and during the pandemic?
- What ongoing lessons are there for multiagency arrangements during and as we emerged from the pandemic to enable effective safeguarding for DVA victims and their children, especially those from Black and minoritized groups?

## Method

This paper draws on the case-study approach adopted as part of the study, as this is an effective methodology to explore complex issues in real-world settings and offers itself to interdisciplinary approaches (Harrison et al., 2017). Two geographically separate case study areas were identified to reflect a microcosm of British society. One area was selected because it is a large, mainly urban conurbation with diverse Black and minoritized communities. The second area was chosen because it is spread over a larger geographic area and comparatively smaller population, comprising both urban and rural areas, is culturally diverse, and has a county structure.

As part of our case-study approach, 15 qualitative interviews with victim-survivors of DVA were conducted, exploring their experiences of negotiating service landscapes during different phases of COVID-19, including experiences of accessing and receiving support. Ethical approval was sought from the university (Ethical Approval 26224) paying specific attention to how we would protect their confidentiality, post-interview safety, and well-being.

Victim-survivor recruitment was via NGO domestic violence providers with support staff identifying potential participants. They excluded those who were too emotionally close to their experience to participate or were in precarious living situations. This method was chosen to maximize victim-survivors safety. The sample (see Table 1) specifically included Black and minoritized women, as these perspectives remain underresearched, and the emerging evidence of a differential impact of COVID-19 on Black and minoritized communities (Khunti et al., 2020).

**Table 1.** Background Details of Participants.

Number of Black or minoritized women	11
Number of women with NRPF	8
Number of White British women	6
Number of women with children	13
Number of women in contact with social services	15
No. of women in contact with police	15
No. of women in touch with health services	8

*Note*. NRPF = no recourse to public fund.

Semistructured interviews took place remotely via SKYPE, Zoom, Microsoft Teams, or telephone due to continuing restrictions around COVID-19 (see Table 2 for topics covered in interviews). Verbal consent was recorded at the start of the interview and interviews were recorded with the camera off, to ensure further confidentiality. A named support worker from the NGO was informed that the interview was taking place when it ended and provided immediate and long-term post-interview support to them to ensure their safety and well-being. The interviews ranged from 45 to 90 min and were carried out by a member of the study team with experience of working with vulnerable service users. All recordings were transcribed verbatim, with two requiring a professional translator (from Urdu to English).

**Table 2.** Topics Covered in Semistructured Interviews of Survivors.

Prior experience/s: Could you share with me, if you want to, the circumstances of the DVA you have experienced? Do you have any dependants? What access for help did you have/any before lockdown?
During COVID-19 lockdown: How did DVA get worse during the lockdown? How did you seek help? From whom? If you didn’t contact the police/nurse (statutory services) why? What helped/did not? If you asked for help from a domestic violence support/NGO, why? If you did not receive a sympathetic response, why do you think this?
After leaving perpetrator: If you remained in the house where you previously lived with the perpetrator, how were you supported by the police/social services, etc.? Did the perpetrator continue to try and contact after you left? How did you cope with this? Did the refuge and statutory services help you? What was your experience in the refuge? What helped least/most?—How did the isolating rules of lockdown make your experience of seeking further help difficult?
Reflection: What do you think would have helped to make you leave the perpetrator easier in lockdown? What would you say to the services in terms of your experience?

*Note*. DVA = domestic violence and abuse.

Three research team members listened to the digital audio recordings of interviews to check the accuracy of the transcripts. Transcripts were also checked to ensure complete anonymization. Regular team meetings were held with the qualitative research team and PI to discuss emerging themes for consistency in the interpretation of data. The data was then analyzed thematically using Clarke and Braun (2013) and employing N-Vivo 12 for data management. Repeated codes were identified by re-reading transcriptions, and re-listening to the recordings. Once these initial codes were identified, themes emerged, which were reviewed, defined, and named.

There was also a quantitative element to the study, which involved analysis of police data in the two case-study areas. Crime-level data for all DVA-flagged cases reported to police for a period prior to the COVID-19 pandemic restrictions in England (August–December 2019), and the same period in 2020, post-lockdown (but still during the pandemic). Variables included victim and perpetrator demographics, risk level, dates/times of reporting, who reported, location, rates, and reasons for incident outcomes at the point of data extraction and referrals to multi-agency safeguarding.

The data provided by the forces included crime records where the crime was reported between the sample dates and where the DVA flag had been used or where a DVA record was attached. Data records obtained from the two forces were subject to data cleaning, which included removing duplicate records and records where either the victim or suspect was aged under 16 years old at the time of the report. Victims aged under 16 were removed as this cohort is not included in the Home Office definition of DVA. The decision was also made to remove suspects who were under 16 years old for comparability. This resulted in a final data set of 29,901 DVA cases from Force 1 and 17,311 from Force 2, with a combined sample of 47,212 DVA cases. Aggregated, anonymized datasets from both forces were analyzed separately by the research teams from the two universities, and overall findings were written up for comparison. For the purposes of this paper, we focus on comparisons between White majority victims and Black and minoritized victims regarding police responses and victim engagement using descriptive and inferential analysis of the police-recorded crime data.

## Findings and Discussion

Our data analysis identified seven key themes emerging from the qualitative data survivor interviews. These interconnected themes showed the additional, manifold layers of complexity and vulnerability for each survivor, challenging their ability to access help. They were: (a) accessing DVA support during COVID-19, (b) trauma, mental health, and finance, (c) no recourse to public funding, (d) impact on children, (e) cultural bereavement and social isolation, (f) service responses (adverse and positive), and (g) survivor recommendations. In almost all cases, the intersecting aspects of COVID-19 restrictions, the continued risk of being found by the perpetrator and reduced resources were more pronounced for Black and minoritized victim-survivors.

Quantitative police data are also brought in throughout the findings section to illustrate and expand themes arising from the interview data, especially to highlight changes in victimization, crime reporting, crime outcomes, and agency responses in the first year of the pandemic (2020) compared with the previous year. As a key focus in this paper is on Black and minoritized victim-survivors, police data were especially analyzed to compare these variables for

Black and minoritized victim-survivors compared to White majority victim-survivors. Victim ethnicity was recorded in 89% of records for one police force and 42% in the other. Where ethnicity was recorded for Force 1, the proportion of victims recorded as Black and minoritized victim-survivors decreased from 16% in Aug–Dec 2019 (pre-COVID) to 13% in Aug–Dec 2020 (post-COVID). Where ethnicity was recorded for Force 2, the proportion of victims recorded as Black and Minority Ethnic (BAME) in Aug–Dec 2019 (pre-COVID) was 7.5%, with a slight increase to 7.7% during Aug–Dec 2020 (post-COVID). Interestingly, Black and minoritized victims recorded higher proportions of adult family abuse categories compared with White victims for both forces.

Table 3 provides key background details of the victim-survivors who were interviewed and the agencies they were in contact with.

**Table 3.** Descriptives From Police Force Data.

	Force 1^a^		Force 2^b^	
Variable	Black and minoritized ethnicity	White ethnicity	Black and minoritized ethnicity	White ethnicity
Breakdown of ethnicity	15%	85%	8%	92%
Suspect–victim relationship				
Current spouse/partner	23%	24%	35%	30.6%
Ex-spouse/partner	39%	44%	32.8%	45.7%
Parent			4.5%	3.1%
Child			10.4%	9.7%
Sibling			10.9%	4.4%
Other			3.8%	4.2%
Unknown			2.6%	2.1%
DASH risk level by victim				
Standard	25.2% (*N* = 267)	22.6% (*N* = 1,448)	42.9% (*N* = 406)	43.1% (*N* = 5,103)
Medium	52.7% (*N* = 558)	54.1% (*N* = 3,464)	41.5% (*N* = 393)	43.6% (*N* = 5,164)
High	22.1% (*N* = 234)	23.3% (*N* = 1,493)	15.6% (*N* = 148)	13.4% (*N* = 1,586)
Average DASH score (*M*)	Not available	Not available	3.14 (*SD* =3.82)	3.6 (*SD* = 4.22)
Crime outcome^c^				
Victim withdraws support	72.5% (*N* = 1,250)	68.1% (*N* = 6,984)	71.4% (*N* = 835)	67.5% (*N* = 9,648)
Process issues	15.4% (*N* = 265)	16.2% (*N* = 1,658)	14.3% (*N* = 167)	13.0% (*N* = 1,852)
Charged/summonsed	7.1% (*N* = 123)	11.2% (*N* = 1,145)	6.2% (*N* = 73)	9.9% (*N* = 1,417)
Other outcomes	5.0% (*N* = 86)	4.5% (*N* = 461)	8.0% (*N* = 94)	9.6% (*N* = 1,368)

a Ethnicity of victim was recorded for 42% of victim records within Force 1 data.

b Ethnicity of victim was recorded for 88% of victim records within Force 2 data.

c Crime outcome percent calculated from all crime outcomes per Force.

### Theme 1: Accessing DVA Support During COVID-19

In analyzing the qualitative data, the quote by Victim-Survivor 1 showed that participants in our study described feeling trapped due to being locked in with their perpetrator:

But then when the lockdown started, there was no way I could avoid it because we were in each other's nose and everything.

The quote reflects the literature, as strict rules of lockdown increased the proximity and vulnerability of victim-survivors with their perpetrators, exacerbating all forms of DVA, and connectedly, reducing the ability for them to seek help (Grierson, 2020; Kaukinen, 2020). External stressors of isolating during lockdowns, such as unprecedented increased unemployment and financial hardships also pushed many households into poverty, resulting in reduced likelihood of victims leaving abusers (Leslie and Wilson, 2020).

For victim-survivors who had managed to connect to services, they faced obstacles leaving or finding a place of safety. Pressures within shelters, the slowing down of services, and an inability to move other victim-survivors to alternative move-on accommodation, reduced spaces for them during COVID-19, as illustrated by Victim-Survivor 12:

… they put me in a refuge, but I was sleeping there on a mattress, on the floor, just for a couple of days, in one of the eating rooms because there were no spaces at all

This experience is solely associated with COVID-19 and would not happen in a nonemergency situation. Associated impacts of accessing help during the COVID-19 pandemic resulted, among others, in homelessness, not being able to access any legal assistance, enforced unemployment due to relocation, and as Survivor 14 described, the inability to access urgent or supported housing, adding layers of complexity for all concerned.

Due to backlog of Covid, I couldn’t access Legal Aid, nor any help to represent me

The experience of victim-survivors reflected, and corroborated difficulties expressed by statutory services and NGOs who act as safeguards for those experiencing DVA. Cortis et al. (2021) referred to the sudden escalation and struggle to assess risk remotely, the reduced mobility of staff within NGOs who support victim-survivors of DVA, and the speed at which practitioners had to adapt and deliver services online (Walklate et al., 2020), accumulating to a confusing service landscape for anyone. Online innovations to enable access to services were often hampered by digital poverty experienced by some victim-survivors, who were often manipulated financially, through isolation, and surveillance (Havard and Lefevre, 2020).

Once in shelters, the restrictions of homeschooling, for example, living in single rooms due to COVID-19, lack of IT equipment, reduced safeguarding protection, and having to change or travel to a new school emerged as complex experiences for both mother and children as illustrated by Survivor 1:

We were going out every day to go to school, it was a long distance because everything doubled, we were really far. I have to change four buses to get them to school, but it was better than the three of us being in one room

For those who could not leave the shelters, pressures were intensified for victim-survivors, often with more than one child, being forced to spend all their time in one allocated room, with access to the outside being limited for official purposes. Survivor 12 stated:

It was a very difficult situation for me especially as I’ve got three children all under five that I have to take care off, all in one room

This is very different from shelter life outside of COVID-19, where children are able to socialize with other children and women, build friendships, give and provide support to other women, and engage in leisure and educational activities to begin to rebuild their lives.

### Theme 2: Trauma, Mental Health, and Finance

Previous studies demonstrate that within DVA support services, over 75% of victim-survivors seeking help have clinical posttraumatic stress symptoms (PTSD), and long-term experiences of depression, anxiety, and other mental health issues (Ellsberg et al., 2008; Ferrari et al., 2016). Similarly, the victim-survivors in the qualitative data reported how abusive and controlling experiences of DVA resulted in a range of mental health and well-being issues for them and their children. They reported a far-reaching sense of unpredictability, helplessness, and disconnection contributed to a range of mental health challenges, including depression, PTSD, suicidal ideations, substance abuse, and anxiety. So, as Victim-Survivor 3 disclosed, while mental health problems are a feature of domestic abuse generally, the pandemic heightened and exacerbated mental health conditions (Esterwood and 2020):

Yes, my life is completely destroyed. I am in mentally torture and mentally stressed … I don’t feel like doing anything. He destroyed my life after twelve years marriage … cheating me … deceived me … I want to do suicide

Anxiety worsened if victim-survivors lacked English language fluency or did not speak the language at all. Heightened anxiety, at being found or harmed by the perpetrator and anxiety about their own and their children's safety were just some of the immediate fears that interfered with providing necessary information to satisfy the police processes (Laxminarayan, 2013). Also, all victim-survivors, such as Survivor 2, expressed the trauma of having to re-tell and relive their story repeatedly to a range of services that required a history of what had happened for their records.

I just want to go to where I get help and I don’t want to come here, tell you my story and then you’re sending me to the next person, I have to tell that same story, you’re just beating me over and over because every time talk about it

Avoiding re-traumatization for survivors is crucial, and it was clear that the services they accessed appeared unaware of repeated trauma endured due to the processes by which services are organized and accessed. Victim-survivors also expressed ongoing trauma of a combination of a lack of financial independence, used previously in their relationship as a form of manipulation, on their mental health: leaving them without (or the know-how to) access funds for food, transport, or taxi fares to take children to school, fears of not being able to afford or pay energy bills or destitution. This worsened with the restrictions of COVID-19. Even when victim-survivors flee DVA, a single parent with limited capacity to earn independently is more likely to report both financial difficulties and ongoing financial abuse from perpetrators such as withholding of child support contributions (Fahmy et al. 2016). As Lloyd (2018) reminds us, it is not uncommon for victims of DVA to risk remaining with perpetrators, rather than jeopardizing themselves, and their children, from becoming homeless. Victim-Survivor 12 illustrates how the lockdown added to this burden, with assistance for financial support worsening due to the slow-down of services:

No, no choice, I depended on my husband for everything … sometimes they [the perpetrator] give you money, sometimes they don’t … then they abuse you; they say they don’t have money. … how can I cope?

In three cases for Black and minoritized women, the welfare benefits previously received were stopped due to (unfounded) suspicion of defrauding the welfare system which impacted their ability to buy food for children or pay for heating. As Victim-Survivor 7 expressed, challenging such decisions in the restrictive lockdown period became impossible to reverse.

I was without electricity for a while, and it was in the winter. I was cold but I had no money, as they had stopped it

For Black and minoritized victim-survivors, this further exacerbated their trauma and mental health issues. Victim-Survivor 4 conveyed staff biases in welfare benefits agencies toward providing minoritized victim-survivors with help.

I think, she thought I was just lying … because of what she said to me, she thought I was part of those Londoner people who normally come with false stories to fraud the system

We argue it is important for services to recognize, respond, and appreciate the extent of psychological trauma experienced by the victim-survivors and their children, exacerbated by COVID-19. This requires joined-up inter-agency collaboration and early referral for psychological support. Su et al. (2022) refer to how greater utilization of alternative ways of psychological support, such as the use of technology-based interventions, has the ability to reduce the strain on waiting lists, but as Burgess (2020) states, prompt intervention of even remote and online mental health interventions needs to be put in place early to improve DVA victims’ long term mental health outcomes. However, the levels of poverty (including digital poverty) may preclude victim-survivors accessing technology-based interventions.

### Theme 3: No Recourse to Public Funding (NRPF)

Where a victim-survivor is subject to the immigration rule of NRPF, they are restricted in being able to access state-funded benefits within the United Kingdom. Reddy and Mahmood (2023) state that NRPF remains a public health risk as it increases the risk of destitution among vulnerable migrants. Significantly, migrant women, who were victim-survivors of DVA and subject to NRPF, were viewed entirely through an immigration lens rather than as victim-survivors of abuse. This worsened during the lockdown with shrinking services and increased pressure on services. Accordingly, these victim-survivors had to face a stark choice: to remain with the perpetrator or leave and risk poverty, isolation, and possible deportation (Voolma, 2018).

Hence, the state, through the creation and maintenance of NRPF, enables perpetrators’ who are UK citizens or have indefinite leave to remain, to control victim-survivors who have NRPF. This was primarily related to deterring the victim-survivor from seeking help, through the threat of deportation, should they end the relationship, thus generating fear in the victim-survivor. Victim-Survivor 5 stated:

… because at the beginning, when I came here, my husband always told me that, the police will never help you because I’m married to you and [not] being a citizen here, it's a problem for you, he did it to make me scared

Alternatively, the perpetrator may threaten to end the relationship which would leave the victim-survivor destitute or isolated, as well as using cultural norms (e.g., the shame of a failed marriage), to silence her and keep her in her place.

Some NRPF victim-survivors faced unhelpful service responses, including from the police, social care, or healthcare staff, who failed to make appropriate referrals to any DVA services. Two examples of this experience are highlighted below by Victim-Survivors 2 and 7:

They [the police] just gave me a piece of paper with a lot of numbers on there and told me to call a solicitor

The midwives and clinic staff, also doctors at ante-natal clinic … would not help me … I kept telling them many times … they claimed they could not help.

Experiences of Victim-Survivors 6 and 3 also made it apparent that these harsh immigration rules not only influenced perpetrators' behaviors but resulted in potentially biased service responses to women who were NRPF.

She contacted many places for me, for refuges. but everybody refused me because I have not access public funds.

So, you are a DV victim, yes, but sorry we cannot support you because you don’t meet our criteria.

Black and minoritized and NRPF victim-survivors found they were not believed at first when reporting abuse and were repeatedly asked testing questions by a range of agencies to verify the truth. This experience is supported by the evidence from Belknap (2010) and Thiara and Roy (2020) who found that Black and minoritized and NRPF victim-survivors who had contact with the police initially, were left feeling disempowered, not feeling heard by having to deal with slow and/or racialized responses that cast them as the problem. Laxminarayan (2013) and Kasturirangan (2008) found victim-survivors who have NRPF often experienced unfriendliness in seeking help because of societal and institutional racism, overt antiimmigrant discrimination, and hostility that allowed professionals to legitimize cross-checking evidence of DVA.

The ongoing manipulation by the perpetrator and/or fears of contacting agencies, hostile treatment by agencies, and potential deportation often resulted in victim-survivors not having the required evidence required by the Home Office, as proof of being a victim of DVA, to aid their quest for indefinite leave to remain in the United Kingdom. Survivor 7 expressed:

So, if I didn't have those evidence to prove that I'd been abused by a British citizen by now, l would be in Nigeria

Additionally, with little financial freedom, the challenges of lockdown, attending or calling the UK Borders Agency (the agency dealing with immigration), with long waiting times, caused further difficulty for these victim-survivors.

Professionals involved in providing support for victim-survivors of DVA seemed to combine a lack of appreciation of the survivor's relative powerlessness to seek help, fears of future isolation or contact with any family, the extent of control some perpetrators adopted to isolate victim-survivors, nor comprehend lack of opportunities victim-survivors experience around available support in an alien country.

For these women, organizational obstacles add another level of bias connected to the unconscious absorption by society of the policing of migration, and the politically perpetuated rhetoric around the abuse of services by “others.” Yuval-Davis et al. (2019) refer to this protective outlook on the nation state's “physical borders” (p.7) which helps to determine and maintain who belongs and who does not. This results in biased policy and service responses to DVA victim-survivors with NRPF, fueled by a “hostile environment” (Ajayi, Chantler and Radford, 2022) including the state-level practice of NRPF. While NRPF has been a long-standing pre-COVID-19 barrier to accessing services, the pandemic worked to exacerbate such difficulties.

### Theme 4: Impact on Children

Victim-survivors with children reported the increased witnessing of DVA due to lockdown measures and the proximity of the perpetrator as disturbing and alarming for the children. Research by Lloyd (2018) and Hughes et al. (2017) link adverse childhood experiences of DVA, with long-lasting effects on physical health, substance misuse, interpersonal violence, and self-harm. For children from all backgrounds, the disappearance of accustomed sociocultural markers exposes them to challenges in identity construction, impedes the development of their personal identity, and affects their psychological well-being (Ventriglio and Bhugra, 2015). Several victim-survivors, such as Survivor 11, talked about the trauma, confusion and potential long-term psychological implications for their children of perpetrator manipulation, for example being treated well by the perpetrator's parent while simultaneously witnessing abuse of their mother.

… he really beat me and the next second, he's ordering pizza for the boys and they’re having fun … the children were so confused

Once they had left the abusive relationship, victim-survivors reported that their children often had ongoing worry about the perpetrator “finding” their mother. This feeling of subjective safety is one that victim-survivors of DVA and their children often continue to report due to uncertainty, apprehension, and anxiety, despite being in a place of safety (Callaghan et al., 2022). Some victim-survivors relayed repeated anxieties from their children. For example, Survivor 8 relayed her children's concern regarding what would happen to them, if any harm would befall their mother or if made by services to go and live with the perpetrator.

Oh, Mommy, if something happened to you, we don't want to go to live with Dad

While the impact of DVA on children is well established in the literature (Holt et al., 2008; Stanley et al., 2010; Stanley, 2011; Hester, 2011), COVID-19 made life harder for children as schools were closed, their normal peer support networks were unavailable, professionals were mostly working on-line which made it harder for children to disclose abuse, all contributing to a heightened sense of isolation and alone-ness heightening the impact of DVA on children.

### Theme 5: Cultural Bereavement and Social Isolation

Eisenbruch (1991) framed and linked the concept of cultural bereavement to the subjective experience of forced migration and PTSD. This concept tends to be discussed in the context of international forced migration, but it was apparent in this study that all victim-survivors, whatever their background, expressed multilevel losses, injustice, and social isolation experienced due to being forced away from their social, psychological, emotional, and geographical location they referred to as home. Their experience of cultural bereavement also related to experiences around the loss of familiar social structures, cultural values, rituals, lack of contact with loved ones, and importantly, a lack of justice for the reason that forced them to move away. According to Burnett and Ndovi (2018) parents and children with previous experience of DVA, are wary about trusting one another and are often reticent about sharing personal information—and this is compounded by a sense of cultural dislocation expressed by Victim-Survivors 9 and 2.

First time in my life I was living outside my home, lonely, with new people, no relations … situation was really bad. I have no idea how kind people are, can I trust them, my language, my religion is different, I don’t belong here.

I thought we would be in London … my first child has support from Speech and Language. an Educational Psychologist … and then there was a sudden change … a decision was made to go all the way to [a different city], which was very difficult moment because we had been moved away from people that supported us. I don’t have a single friend here.

It is apparent that this concept can clearly be linked to the interconnected subject of mental health, but it stands apart because it allows for understanding and appreciating the experiences of victim-survivors as complex and multilayered for those forced from their idea of home.

### Theme 6: Service Responses

This theme has been divided into two areas: adverse responses and positive responses expressed by the victim-survivors regarding those who have responsibility for supporting them.

#### 6A: Adverse Responses

It was apparent that some victim-survivors reported a lack of understanding by professionals in statutory services about the definition of DVA used in England and Wales. Some described adult family abuse as being poorly understood despite it being part of the definition of domestic abuse in England and Wales. For instance, as Victim-Survivor 15 described, there was a lack of referral for support for adult family abuse between siblings:

I contacted the police, one week … 6 times, but because he was my brother, they said it was a ‘domestic matter’ or ‘sibling rivalry’, even when he was punching, beating me, saying that he wanted to kill me. Social services nor housing association claimed that they could not do anything. Despite attempting suicide, my GP did not refer me to the mental health services

Although the police force reported that during the pandemic, they had less to do with other crimes and were able to respond quicker and more thoroughly, some victim-survivors still felt that the police had not responded in a timely fashion. One victim-survivor (14) described the police not attending a home visit in response to her call, and having to wait several days, despite a protection order being in place:

Despite violating access to me, including physical violence, no one took action. I called the police repeatedly … no action … they didn’t come for days

The most widely used risk assessment tool utilized by the police in England and Wales is the Domestic Abuse Stalking and Honour-based violence (DASH) tool. Depending on how many factors are present in each DVA situation, a risk rating of standard, medium, or high is given, but the final risk rating is also influenced by professional judgment. Our police quantitative data analysis revealed that similar proportions were recorded at each DASH risk level for White and Black and minoritized victims of DVA for both force areas. However, there was a significant effect of victim ethnicity on the number of DASH risk factors ticked, indicating that Black and minoritized DVA victim-survivors (*M* = 3.14/*SD* = 3.82) had significantly less DASH risk factors ticked compared to White DA victim-survivors (*M* = 3.60, *SD* = 4.22). This suggests that, overall, Black and minoritized victim-survivors may have had a less accurate DVA risk assessment than White victim-survivors. Similarly, Black and minoritized victims were significantly less likely to have a charge brought against their perpetrator and had significantly more cases with the outcome “Victim Withdraws Support” than White victims. While the reasons for these disparities are not discernible from the quantitative data, the qualitative data offers insight into the difficulties of accessing support from the perspective of Black and minoritized victim-survivors.

There seemed to be a lack of appreciation from some staff, across all sectors, around the victim-survivors’ levels of risk and trauma and the extent to which perpetrators would go to manipulate and threaten the survivor. In one instance, the perpetrator with a legal injunction to stay away from his wife, baby, and property was facilitated by the police to return to the house to collect more of his property. He had previously threatened to kill his wife and newly born baby. The police officers, however, according to Survivor 3, kept focusing on the survivor's need to be calm, despite his threats to her and her child:

So, I was crying in the room and the lady … because it was one officer and a lady officer … she told me to calm down, but I was with my 2-month-old baby and he was speaking French loud just for me to understand, the police doesn’t understand French, and he's threatening me in French!

Several victim-survivors reported that some professionals, including the police and midwives, were challenging to deal with. Other than the issue of trust, some said that they experienced male police officers advising victim-survivors to seek help themselves, as expressed by Survivors 7 and 4, rather than concentrate on removing the perpetrator:

He was still coming all the time, to disturb me, bang on my door, just wait for me outside … I had to call the police, on several occasions, it was always men only coming, telling me whom to call, they never removed him.

When I was giving birth, he managed to come to the ward and would stand outside the ward. The staff knew about him…But he created confusion … they almost let that happen, he would have been able to come to my room while I had the baby.

The experiences and stories relayed by victim-survivors who had adverse responses indicate that professionals in statutory services need to improve their understanding and responses to domestic abuse. Some victim-survivors expressed dismay when disclosing to professionals who tended to focus only on obvious acts of aggression and violence but ignored the range of micro-aggressions, micromanagement, and isolation (coercive control) of women and children's everyday lives. The accounts illustrated the importance of understanding DVA encompassing diverse forms of abuse.

#### 6B: Positive Responses

Importantly, some victim-survivors also experienced positive support to protect and keep them safe. For several victim-survivors, it was the National Health Service staff (midwives, nurses, general practitioners), who had instigated help, pointing out that what they were experiencing was coercive control, provided leaflets, contacted social services, or put them in touch with groups that dealt with DVA. One midwife even admitted Victim-Survivor 2 into the hospital as a way of ensuring that she received the protection she needed, despite the survivor having NRPF:

I had the NHS support because I was in a high-risk pregnancy. I was really looked after very, very closely by the whole NHS staff because of this … they had suspicions because of my health issues, so they kept me in purposely.

Social services professionals also, in many instances ensured they arranged and organized the completion of application forms, not just for the survivor but also for their children. In the case of several NRPF women, social workers ensured they had access to specialist immigration solicitors who could support them. Several participants, such as Victim-Survivor 7, talked about how their Social Worker had supported above and beyond the role expectations:

She actually made quite a lot of arrangement for me. She applied to Sure Start for the children, connected me to solicitors for immigration, solicitors to handle anything concerning the children because of their father.

Victim-Survivor 15, who was experiencing threatening behavior from her brother and previously ignored by police, stated it was one police officer, who eventually heard her and drove her directly to a shelter for support.

He took me, what little I had and drove me to a place out of town, to a refuge.

Several victim-survivors, such as Victim-Survivor 9, who had NRPF found the police (both male and female) who eventually rescued them, were overcome by how kind and warm they were, having been told by the perpetrator that no one in this country would support them:

When I was in the police station, I was crying and at the time nobody can touch each other but the lady … she just hold me, showing me you are secure, you are safe, don’t worry and she just hold me in her arms.

Support came from a wide range of professionals including a university lecturer such as for Victim-Survivor 1:

My biggest support was my uni, were morally supportive, physically supportive … financially supportive … I would say I had it covered from uni 100%.

Once safe in the shelters, although restrictions were in place, all victim-survivors, such as Victim-Survivor 9, talked about the time, and space they were given to recover and the ongoing support they were receiving.

They arranged me some mental health services, at that time I’m not speaking English, but they listen. I need to cry, they listen. Even I don’t think they understand … I am speaking and crying, they believe and listening me.

### Theme 7: Survivor Recommendations

Several victim-survivors recommended the importance of having ethnically and linguistically diverse staff in key organizations so that they are able, not just to understand the language but the cultural nuances of the language and the meaning attached to specific words or phrases. As Survivor 1 explained, this would reduce misunderstandings, for example, in the ways English is expressed in different cultural contexts:

They should have people of different backgrounds into those key organisation places because they understand the victim from the cultural point of view, even in the way we may use English differently

Those victim-survivors who had NRPF felt that there needed to be more creative ways and education for women who did not have ways of accessing help in the “normal” way. Survivor 4 felt that the use of community centers and groups, to leave information, in toilets and in prayer spaces were some of the suggestions put forward.

There could be more education because more women are indoors, suffering especially those who do not have paperwork. They’re scared. Pinpointing those areas that we can go secretly to talk to about what's going on. Show more ways of escaping … I got to know escaping through my health visitor.

Several victim-survivors talked about the importance of psychological support or counseling early on, be it individual or family-based counseling. One victim-survivor felt that it was an investment to reduce future issues, such as self-harm, and to support and educate children from normalizing the abusive behavior that they had experienced. She stated that the confidence she developed from being heard was unmeasurable.

Victim-Survivor 1 talked specifically about the importance of talking to young immigrant men about what was acceptable behavior with women. She expressed that the loss of status for immigrant young men in their new country could often result in them acting out more masochistic behaviors than they may have witnessed in their country of origin (see Alesina et al. (2016). She reported that unless measures were put in place to educate young men, then the process of removing victims and would-be victim-survivors from DVA would continue:

If you have a seminar, tell them this is wrong … so, either you want to spend money taking care of these women, putting them in hostels, or you try to solve the problem from the root … he is still who he is, the next woman will be the next victim.

## Conclusion

The analysis of survivor voices provides a significant opportunity for policymakers and professionals in safeguarding roles to build on areas of good practices mentioned and to develop sensitive service responses to address the challenges identified. As suggested by Kulkarni et al. (2012) and End Violence against Women (EVAW) coalition (2022), those researching perspectives from DVA victim-survivors need to recognize that accepting the specific experiences and needs of the differently situated victim-survivors is crucial for planning more person-focused, person-centered policy resolutions. Our focus on Black and minoritized victim-survivors within the pandemic seeks to precisely do that and this conclusion will consider the key practice recommendations and the learning from this study.

In considering the experiences of victim-survivors accessing support during COVID-19, the pandemic has highlighted existing inequalities and exacerbated them. Krishnadas and Taha (2020) argue what the pandemic exposed was the lack of adequate funding and shelter provision in place for women fleeing DVA generally. It has brought into sharp relief the intersections of VAWG, structural racism, and institutional racism (or the “multiplier effect”), which have made accessing support particularly difficult for Black and minoritized women. The multiplier effect was most visible in relation to NRPF—a major barrier to accessing services for Black and minoritized victim-survivors. NRPF has also been identified in previous research (Odumade and Graham, 2019; Speed et al., 2020) as a challenge, but this study shows how NRPF intensified the challenges to accessing support during the pandemic as shelter spaces were limited, and there was very little move-on accommodation available—in addition to the logistics of moving during the pandemic. NRPF results in multilevel challenges faced by these victim-survivors, including destitution and the increased capacity for the perpetrator to manipulate the survivor with the threat of deportation, and the amplified struggle for the survivor fleeing the abusive situation. It should be noted that deportation remains a possible outcome if a woman with NRPF leaves an abusive relationship with a British citizen or a person with indefinite leave to remain. Despite the ongoing campaign by several women's organizations to abolish NRPF, particularly in the lead-up to the Domestic Abuse Act (2021), this has not happened. Abolishing NRPF would be an optimistic recommendation and remains a cherished hope of protecting and supporting victim-survivors subject to this rule. The main learning here remains that those involved in providing help to victim-survivors should be aware of the extent to which it suppresses and impacts help-seeking, while at the same time emboldening controlling perpetrator behavior.

Our findings, in considering the impact on Black and minoritized victim-survivors, also confirm that the NRPF status contributed to perpetuating hostility or suspicion among professionals, whereby victim-survivors were stereotypically constructed as not entitled to any services or as potentially abusing the system to remain in the country (Kallivayalil, 2010). Fundamentally, the ask is for the provision, responses, and collaboration of DVA safeguarding services to be based on the principle of social equity, encompassing cultural sensitivity, and understanding the multiple layers of disadvantage that women with NRPF experience. Services responses need to be enhanced by demonstrating greater empathy and sensitivity to victim-survivors, and within the context of our findings, specifically to those with low income, Black and minoritized victim-survivors and those with NRPF. While this reflects what was known pre-COVID, the ‘multiplier effect’ of the pandemic makes it even more urgent to abolish NRPF. We concur with the UK's Domestic Abuse Commissioner (Safety Before Status; 2021) that at the very least there should be a firewall between victim-survivors with NRPF and the UK Border Agency (UKBA) so that accessing support does not automatically mean that the UKBA will know of the victim-survivors whereabouts—thus increasing confidence in coming forward to access support and limiting the likelihood of deportation.

In considering what were the police responses to Black and minoritized women pre and during the pandemic, victim-survivors reported a mixed response from a range of professionals: the police, social workers, and healthcare professionals which included homophobia, sexism, racism, ethnocentricity, and assumptions about a lack of intelligence of victim-survivors. The police data shows that ethnicity recording needs to be improved overall and made more consistent between forces and that Black and minoritized victims received more adverse crime outcomes, with higher victim withdrawal rates and lower numbers of DVA crimes charged/summonsed. One key learning here was that there was a significant effect of victim ethnicity on risk assessment with Black and minoritized victim-survivors having significantly less DASH risk factors ticked compared to White victim-survivors. It is not possible to discern why this might be the case from the quantitative data, but the qualitative analysis indicates some possible explanations (e.g., language and cultural fluency; not fully appreciating the vulnerability and position of women with NRPF, insufficient understanding of adult family abuse). However, variations in ethnicity recorded by police highlight an urgent need to further investigate the differences in risk assessment recordings made by police officers and an increased understanding of the nature of DVA offending. It is recommended that there is a generalized need for those involved in supporting victim-survivors of DVA to have an increased ongoing understanding and open discussions that are embedded in policy, regarding how systemic racism, other forms of discrimination, and unconscious bias of professionals can impact service responses to DVA victim-survivors.

It is incumbent to learn from the examples of good practice and to challenge unhelpful responses so that victim-survivors and their children are offered the best protection possible. The variability in response to victim-survivors, especially toward Black and minoritized women, leaves too many gaps in services, and a more uniform, positive response is required across all services. This has implications for individuals’ professional practice as well as the need for organizational systems and structures to enhance, develop, and support antidiscriminatory professional practice.

Survivor experiences strongly allude to the need for recurring training, awareness, and consciousness on the part of policymakers and stakeholders, to make services relevant to the complex, diverse, intersectional experiences of victim-survivors. This recommendation should be supported with regular updates that also need to focus on work toward improving understanding of the definition of DVA, so that the complex experiences of DVA are better understood and responded to, thus ensuring survivors are not left in harm's way.

Alongside a decade of austerity measures, the uncertainty and unpredictability of COVID-19 has exposed multiple issues, with human and economic impacts on DVA victim-survivors. For families scrutinized and impacted by immigration conditions, this scenario is worsened by the associated ongoing extreme poverty and precarity they have already been facing (Dickson, 2022) contributing to psychological and mental health issues that require interventions from a range of services that should work toward a more survivor focused collaboration. As such, in considering the ongoing lessons from multi-agency arrangements, especially for Black and minoritized groups, we recommend that there is a need for increased interdisciplinarity and collaborative interconnections (with associated funding) between mental health services and all those groups who potentially come across and support victim-survivors of DVA. Backed up with increased government funding, alliances also need to be made with Black and minoritized community-based NGOs, who were reported as being more culturally sensitive and sympathetic to the intersectional experiences of the victim-survivors.

Extant literature discusses the impact of DVA on children and was replicated in our study, worsening in the pandemic. Previous studies (Antle et al., 2010; Curran, 2013) have revealed the long-term effects on the behavior of children who experience the manipulative behavior of a parent who perpetrates DVA. Thus, as recommended above, the provision of safety and support for survivor children alongside the survivor parent requires early, multidisciplinary, collaborative interventions. This study sheds light on how future planning for pandemics or emergencies should ensure that safeguarding women and children from DVA is highlighted as central to emergency planning. The increased need for collaboration between child protection services, adult statutory services, the police, and the independent domestic violence sector was apparent as DVA often takes place outside the child protection arena. Within this planning, the ongoing lessons are that it is essential that statutory services (and the associated funding from the government), anticipate, collaborate, and prepare for: increases in DVA, the vulnerability of victim-survivors, an understanding of the complex multiplier effect on Black and minoritized victim-survivors, and that such plans and policies be driven by an intersectional approach so that the needs of the range of marginalized and minoritized groups are taken into account.

Fundamentally, the key practice recommendations and learning from this research should start with understanding how essential victim-survivor voices are and remain to future development of DVA safeguarding so that services are truly fit for purpose. This, in itself, has the double-edged benefit of empowering victim-survivors (Goodman et al., 2016). Practices defined by victim-survivors in this research have shown that responses that promote client choice, partnership, and a sensitivity to the intersectional needs, contexts, and coping strategies of individuals can empower victim-survivors. This approach should allow all stakeholders involved in DVA to learn from, develop, and put in place policies to help consider those practical considerations such as lack of funds, anxieties around linguistic fluency, cultural differences, the silencing effects of DVA, the impact of abuse on self-esteem, stigma, and retraumatization alongside systems of power and inequity that stop those experiencing DVA even attempting to seek help in the first place.
